# Predictive power of UKCAT and other pre-admission measures for performance in a medical school in Glasgow: a cohort study

**DOI:** 10.1186/1472-6920-14-116

**Published:** 2014-06-11

**Authors:** Nana Sartania, John D McClure, Helen Sweeting, Allison Browitt

**Affiliations:** 1 University of Glasgow School of Medicine, Wolfson Medical School Building, University Avenue, G12 8QQ Glasgow, UK; 2 Institute of Cardiovascular and Medical Sciences, Glasgow, UK; 3 MRC/CSO Social & Public Health Sciences Unit, Glasgow, UK; 4 Recruitment and International Office, University of Glasgow, Glasgow, UK

**Keywords:** UKCAT, Predictive validity, Widening participation, Socio-economic indicators, Admissions interview, School HE participation rate

## Abstract

**Background:**

The UK Clinical Aptitude Test (UKCAT) and its four subtests are currently used by 24 Medical and Dental Schools in the UK for admissions. This longitudinal study examines the predictive validity of UKCAT for final performance in the undergraduate medical degree programme at one Medical School and compares this with the predictive validity of the selection measures available pre-UKCAT.

**Methods:**

This was a retrospective observational study of one cohort of students, admitted to Glasgow Medical School in 2007. We examined the associations which UKCAT scores, school science grades and pre-admissions interview scores had with performance indicators, particularly final composite scores that determine students’ postgraduate training opportunities and overall ranking (Educational Performance Measure - EPM, and Honours and Commendation – H&C). Analyses were conducted both with and without adjustment for potential socio-demographic confounders (gender, age, ethnicity and area deprivation).

**Results:**

Despite its predictive value declining as students progress through the course, UKCAT was associated with the final composite scores. In mutually adjusted analyses (also adjusted for socio-demographic confounders), only UKCAT total showed independent relationships with both EPM (p = 0.005) and H&C (p = 0.004), school science achievements predicted EPM (p = 0.009), and pre-admissions interview score predicted neither. UKCAT showed less socio-demographic variation than did TSS.

**Conclusion:**

UKCAT has a modest predictive power for overall course performance at the University of Glasgow Medical School over and above that of school science achievements or pre-admission interview score and we conclude that UKCAT is the most useful predictor of final ranking.

## Background

Traditionally, it has been accepted that academic criteria should play a major role in the Medical School selection process, as these best predict outcomes [1-5]. However, as the number of academically able applicants is high and available Medical School places relatively few, additional criteria are needed to identify candidates most suitable for the medical profession [6-8]. A number of medical educationalists rate non-teachable, non-cognitive traits particularly highly and call for higher weighting for these in the admissions process [9-11]. These traits are generally evaluated via admissions interviews, although these have been criticised for a perceived lack of reliability [7,12], predictive validity [13,14] and for the potential bias through preconceptions and prejudice [15-17], all of which could lead to the unfair exclusion of certain groups based, for example, on gender, ethnicity or social background.

In 2006 a consortium of Medical Schools jointly developed the UK Clinical Aptitude Test (UKCAT), designed to test aptitude and various reasoning and intellectual abilities rather than academic knowledge as assessed by school exams. Most importantly, the test was believed to have the potential ‘to improve fairness in the system’ [18,19] and widen participation of non-traditional applicants from disadvantaged backgrounds by reducing the influence of selective schooling [20]. This goal has been promoted by successive British governments through various schemes and initiatives.

As currently used, the UKCAT mainly measures cognitive skills via four sub-scores (verbal reasoning, quantitative reasoning, abstract reasoning and decision analysis), and each Medical School utilises the test results in different ways to augment their own admissions process [21].

Since its inception, several research papers have been published on the use of the UKCAT and its relationship with course performance. Early research papers from single or two-site studies provided conflicting results [22-25] about its predictive value, but a recent (2013) multi-centre study has clearly identified that the test predicts year 1 exam results in 12 Medical Schools and that, when previous educational attainment and a wide range of socio-economic indicators are taken into account, it does add value to the selection process [2].

To test whether this conclusion is generally applicable to other medical schools, several longitudinal studies of students’ performance throughout the entire course were initiated, but because medical students study for 5–6 years, it is only now that studies of the potential predictive validity and practical value of the UKCAT are beginning to emerge.

The Glasgow Medical School adopted the test in 2007 and applicants who achieved the highest UKCAT scores (first quartile) were allocated extra credit (equivalent to 5% of total) in addition to an overall interview score (94%) and any additional credit for exceeding the minimal academic entry requirements (1%). Together, these formed the basis for offer of admission. Analysis of the final outcomes for the first ‘naïve’ cohort to have taken the test without any prior knowledge of it, and the extent to which UKCAT added value to traditional admissions criteria is of great importance for understanding the test performance.

With this objective in mind, the primary objective of the current analysis, based on the results of the Glasgow Medical School 2007/2008 academic year intake was to investigate:

• how UKCAT (total and sub-scales) and other pre-admissions criteria (school science achievements and interview score) were associated with final course outcomes, both individually and in mutually adjusted analysis, both with and without adjustment for potential socio-demographic confounders.

Secondary objectives were to examine:

• Socio-demographic variations in the pre-admissions criteria;

• Associations between UKCAT and the two other pre-admissions criteria used before the test was developed; and,

• Associations between the pre-admissions criteria and years 1 and 5 course performance indicators.

## Methods

### Sample

This was a retrospective observational study of one cohort of students admitted to Glasgow Medical School in 2007–2008. UKCAT scores, school science grades and admissions interview scores were compared with final performance indicators (and with years 1, 5 written and clinical exams and coursework); data included self-reported demographics.

Students were eligible for inclusion in the study if they had taken the UKCAT test and provided written opt-in consent for the use of their admissions and performance data. The cohort consisted of 243 students, out of whom 31 were exempt from UKCAT (21 who applied in 2006–2007 and deferred their entry; 4 transfers from science degrees; 6 returning to the course). The remaining 212 were asked to provide consent for their data to be used in the study and 189 (89%) did so. Attrition rates throughout the course were extremely low (~1%). 9 students who repeated the year at some point during the course and the 51 intercalating students, who re-joined the medical programme after their BSc, were included as we allowed 6 years for completion (the standard medical degree is 5 years).

As reported in Table 1, 56% of the sample were female, 68% were aged 18 or less at admission and 82% were of White ethnicity; most were from less deprived areas (69%) and had one or both parents with higher educational qualifications (81%). Half had attended Scottish schools with a high progression to higher education, compared with only 15% from Scottish schools with low progression rates, with the remainder coming from elsewhere in the UK (25%) or overseas (10%).

**Table 1:** Bivariate associations (ANOVA) between pre-admission measures* according to socio-demographic characteristics mean scores for each group *(and significance)*

	***(N)***	***(%)***	**UKCAT-Total score**	**UKCAT –Verbal reasoning**	**UKCAT –Quant reasoning**	**UKCAT–Decision analysis**	**UKCAT –Abstract reasoning**	**Total Science Score**	**Interview score**^#^
**Gender**									
Male	***(83)***	*(43.9)*	0.096	0.012	**0.251**	0.021	-0.016	**0.196**	0.069
Female	***(106)***	*(56.1)*	-0.076	-0.009	**-0.197**	-0.016	0.012	**-0.152**	-0.054
*(sig)*			*(0.242)*	*(0.885)*	***(0.002)***	*(0.800)*	*(0.848)*	***(0.018)***	*(0.403)*
**Age**									
18 or less	***(128)***	*(67.7)*	**0.100**	0.051	**0.149**	0.012	0.068	**0.316**	-0.034
Over 18	***(61)***	*(32.3)*	**-0.210**	-0.107	**-0.313**	-0.025	-0.142	**-0.691**	0.071
*(sig)*			***(0.047)***	*(0.312)*	***(0.003)***	*(0.811)*	*(0.178)*	***(0.000)***	*(0.504)*
**Ethnicity**									
White	***(153)***	*(81.8)*	**0.093**	**0.093**	0.050	0.052	0.054	-0.056	0.058
Non-white	***(34)***	*(18.2)*	**-0.397**	**-0.435**	-0.205	-0.188	-0.240	0.289	-0.269
*(sig)*			***(0.010)***	***(0.005)***	*(0.180)*	*(0.207)*	*(0.123)*	*(0.080)*	*(0.086)*
**Deprivation**									
Lower	***(130)***	*(68.8)*	0.026	-0.021	-0.014	**0.057**	0.043	-0.023	-0.031
Higher	***(32)***	*(16.9)*	-0.326	-0.052	-0.238	**-0.394**	-0.159	-0.130	0.159
Missing	***(27)***	*(14.3)*	0.260	0.165	0.349	**0.192**	-0.019	0.263	-0.037
*(sig)*			*(0.070)*	*(0.644)*	*(0.076)*	***(0.040)***	*(0.590)*	*(0.306)*	*(0.616)*
**Parental HE**									
No	***(34)***	*(18.8)*	-0.053	-0.007	-0.035	-0.057	-0.041	**-0.712**	-0.125
Yes	***(147)***	*(81.2)*	0.040	0.037	0.027	0.023	0.020	**0.125**	0.046
*(sig)*			*(0.628)*	*(0.815)*	*(0.748)*	*(0.679)*	*(0.754)*	***(0.000)***	*(0.372)*
**School HE participation**									
Scotland – high	***(96)***	*(50.8)*	0.109	0.056	0.111	0.125	-0.013	**0.044**	0.003
Scotland – low	***(28)***	*(14.8)*	-0.240	-0.043	-0.140	-0.323	-0.108	**-0.034**	-0.122
Overseas	***(18)***	*(9.5)*	-0.232	-0.484	0.037	0.095	-0.308	**0.677**	-0.332
Rest of UK	***(47)***	*(24.9)*	0.010	0.097	-0.158	-0.098	0.208	**-0.313**	0.194
*(sig)*			*(0.295)*	*(0.171)*	*(0.403)*	*(0.169)*	*(0.253)*	***(0.007)***	*(0.245)*
**Graduate**									
No	***(146)***	*(77.2)*	0.061	0.001	**0.117**	0.006	0.050	**0.317**	-0.029
Yes	***(43)***	*(22.8)*	-0.208	-0.004	**-0.398**	-0.020	-0.170	**-1.150**	0.098
*(sig)*			*(0.120)*	*(0.973)*	***(0.003)***	*(0.881)*	*(0.207)*	***(0.000)***	*(0.466)*

*All pre-admission measures standardised as z-scores.

^#^Interview score transformed to reduce skew prior to standardisation.

We compared the study and non-study groups and found that the study group is representative of the total cohort in terms of the above socio-demographic characteristics. In addition, the overall admissions points averages for the two groups were identical (84 points).

### Measures

Data were provided by UKCAT and the Universities and Colleges Admission Service, UCAS (the organisation responsible for managing applications to higher education courses in the UK) and extracted from the University of Glasgow admissions and student record system. Examination marks for each year of the course were collected, collated in Microsoft Access and transferred to SPSS v21 for analysis.

#### Pre-admission measures

These comprised the UKCAT total and each sub-score: verbal reasoning, quantitative reasoning, abstract reasoning and decision analysis, each scored out of maximum 900. As a measure of prior academic attainment, the average tariff score for school science subjects, described here as total science score (TSS) was calculated using the UCAS tariff points scale, as described by Yates & James [22]. Scottish Highers are assigned 80 points for grade A and 65 points for grade B and Advanced Highers 130 points for grade A and 110 points for grade B, A-levels receive 120 points for an A grade and 100 points for a B; lower grades are allocated fewer points. Interview scores (out of 84) were given by two independent admissions tutors following a semi-structured admissions interview. The questionnaire used during the interview aimed to ascertain whether candidates considered the implications of a medical career, were informed about the course structure, displayed the characteristics desirable in a future doctor and demonstrated commitment and motivation for a medical career (sample available as an online appendix: Additional file 1.pdf).

#### The course and its performance indicators

The medical degree in Glasgow is a systems-based integrated course with a large Problem-Based Learning component. The first two years of the programme cover related biomedical sciences of the major clinical systems and in years 3–5 students are taught through clinical systems, with a focus on pathophysiology.

Performance is recorded at the end of years 1, 2, 3 and 5, for each of the three separate assessments (written, coursework and a form of clinical examination).

At the end of year 4, the composite Educational Performance Measure (EPM) score is calculated for each student. It is based on exam scores up to this point, and includes student selected components (SSC) and, optionally, an intercalated BSc programme. This score determines the chance of acquiring the first choice training post within the NHS (Foundation Training Programme). After the Finals in year 5, the Honours and Commendation (H&C) composite scores are calculated to determine students’ final ranking, based on overall performance in the course but weighted towards the final year.

EPM and H&C are the primary outcome measures in this paper.

#### Socio-demographic and other individual measures

Variables representing student *gender*, *age at entry* (categorised for the purpose of analysis as 18 years or less versus older) and *ethnicity* (categorised as white versus non-white) were included. *Area deprivation* was identified as the relative socio-economic deprivation of home postcode via the (Scottish & English) indices of multiple deprivation [26,27]. These were grouped into three categories: more deprived (the 40% most disadvantaged postcode areas), less deprived, and a small number of those from outwith the UK, grouped with students whose postcode data were missing. *Parental educational qualifications*, as self-reported during the UCAS application, were categorised as higher education versus none. In addition, student’s *school higher education (HE) participation* rate was categorised into one of four categories: Scottish schools with high participation (>32% of school leavers progress to HE – representing the Scottish average rate over 3 years, 2007–2009); Scottish schools with low participation (≤32% progression to HE); schools from the rest of the UK; and finally, a small group from schools outwith the UK or where these data were missing. Finally, students were defined as *graduate entry* (versus non-graduate), based on previous higher education qualifications.

### Data analysis

Analyses were conducted using IBM SPSS-21. Preliminary analyses showed skewed distributions (skew statistic > 1.0) for one pre-admissions measure (interview score) and two of the outcome measures (Composite H&C and year 5 written exam). Transformation reduced these to acceptable levels (exponentiated interview score, skew statistic = 1.00; square root of Composite H&C, skew statistic = 0.74; square of year 5 written exam score, skew statistic = -0.79). Following this, all pre-admission measures and course performance indicators were standardized to z-scores (mean = 0, standard deviation = 1) for the purpose of analysis. This was both to facilitate comparison of associations and because the wide range of some performance measures might otherwise result in regression coefficients which were highly significant but very small.

The significance of differences in the pre-admissions measures according to the categorical socio-demographic variables, were determined via ANOVA. Associations between UKCAT (total and sub-scores) and the other two preadmissions criteria (TSS and interview score) were determined via Pearson’s correlations.

The SPSS General Linear Modelling (GLM) procedure was used to carry out multiple regressions to determine associations between the pre-admissions measures and course outcomes. First, bivariate relationships between each pre-admissions measure and course outcomes were determined. Our use of z-scores for both independent and dependent variables in these analyses means that the regression coefficients resulting from the analyses were standardised (SPSS GLM output does not include standardised regression coefficients). Second, these analyses were repeated, with adjustment for socio-demographic confounders. Expected associations between the socio-demographic variables (see Additional file 2: Table S1) together with relatively small sample size meant that a restricted set of confounders was chosen (gender, age, ethnicity and deprivation).

Next, in order to examine the independent effects of UKCAT, TSS and interview score, multiple regression models entered all three (i.e. mutually adjusted associations) in respect of each course outcome. Finally, we examined the independent effects of UKCAT, TSS and interview score after adjustment for socio-demographic confounders. All multiple regression analyses reported here entered all independent variables in one block.

All multiple regression analyses were conducted on those with complete data on all four confounder variables (n = 187).

## Results

### Associations between pre-admissions measures and socio-demographic characteristics

Table 1 shows how UKCAT, TSS and interview scores (all standardiased as z-scores) were patterned according to gender, age, ethnicity, deprivation, parental higher education, school higher education participation and graduate status.

UKCAT showed less socio-demographic differentiation than did TSS. Interview score was not significantly associated with any socio-demographic measure. UKCAT total score differed significantly only in respect of age and ethnicity (higher scores among younger and white entrants). Among the sub-scores, abstract reasoning showed no socio-demographic differences, whereas verbal reasoning was significantly higher among white entrants, and decision analysis scores were lowest among those from higher deprivation. UKCAT quantitative reasoning showed the greatest number of socio-demographic differences of all the sub-scores, being significantly higher among males, younger and non-graduate entrants. TSS was significantly higher among males, younger entrants, those whose parents had received higher education, those from overseas, and non-graduates.

### Associations between pre-admissions measures

Table 2 shows how UKCAT was associated with the other pre-admissions criteria. There were only two significant associations; quantitative reasoning was positively associated with TSS and decision analysis was negatively associated with interview score.

**Table 2:** Bivariate associations (correlations) between UKCAT (total and subscales) and both total science score and interview score*-Pearson’s r (*and significance)*

	**Total science score**		**Interview score**^**#**^	
	**Pearson’s r**	***(sig)***	**Pearson’s r**	***(sig)***
**UKCAT – Total score**	0.095	*(0.197)*	-0.030	*(0.686)*
**UKCAT – Verbal reasoning**	-0.054	*(0.464)*	0.007	*(0.922)*
**UKCAT – Quantitative reasoning**	**0.265**	***(0.000)***	0.085	*(0.244)*
**UKCAT – Decision analysis**	0.028	*(0.701)*	**-0.161**	***(0.027)***
**UKCAT – Abstract reasoning**	0.029	*(0.693)*	0.018	*(0.801)*

*All measures standardised as z-scores.

^#^Interview score transformed to reduce skew prior to standardisation.

### Associations between pre-admissions measures and final course outcomes

Our primary objective was to investigate how UKCAT and other pre-admissions criteria were associated with the two final (composite) course outcomes. Table 3 shows the bivariate associations which UKCAT total, its sub-scores, TSS and interview score had with EPM and H&C, both unadjusted and after adjustment for socio-demographic confounders (gender, age, ethnicity and deprivation).

**Table 3:** Bivariate associations (multiple regression analyses) between each pre-admission measure and composite course performance indicators* in models with and without adjustment for confounders – beta (95% confidence intervals, significance) and R^**2 **^**of unadjusted model**

	**Educational performance measure**		**Honours & commendation**^**#**^	
	**Unadjusted**	**Adjusted^**	**Unadjusted**	**Adjusted^**
**UKCAT – Total score**				
Beta (95% CIs)	**0.216 (0.074-0.357)**	**0.212 (0.067-0.358)**	**0.251 (0.110-0.392)**	**0.217 (0.070-0.364)**
*(sig)*	***(0.003)***	***(0.005)***	***(0.001)***	***(0.004)***
*R*^*2*^	*0.047*		*0.063*	
**UKCAT – Verbal reasoning**				
Beta (95% CIs)	**0.213 (0.071-0.354)**	**0.219 (0.076-0.361)**	**0.201 (0.059-0.344)**	**0.170 (0.024-0.315)**
*(sig)*	***(0.003)***	***(0.003)***	***(0.006)***	***(0.023)***
*R*^*2*^	*0.046*		*0.041*	
**UKCAT – Quantitative reasoning**				
Beta (95% CIs)	**0.219 (0.079-0.360)**	**0.237 (0.089-0.385)**	**0.216 (0.074-0.358)**	**0.205 (0.054-0.356)**
*(sig)*	***(0.002)***	***(0.002)***	***(0.003)***	***(0.008)***
*R*^*2*^	*0.049*		*0.047*	
**UKCAT – Decision analysis**				
Beta (95% CIs)	0.125 (-0.020-0.270)	0.097 (-0.049-0.243)	**0.174 (0.030-0.319)**	0.141 (-0.006-0.288)
*(sig)*	*(0.091)*	*(0.192)*	***(0.018)***	*(0.059)*
*R*^*2*^	*0.016*		*0.030*	
**UKCAT – Abstract reasoning**				
Beta (95% CIs)	0.014 (-0.131-0.159)	0.012 (-0.132-0.156)	0.074 (-0.072-0.219)	0.053 (-0.093-0.198)
*(sig)*	*(0.849)*	*(0.869)*	*(0.319)*	*(0.476)*
*R*^*2*^	*0.000*		*0.005*	
**Total science score**				
Beta (95% CIs)	0.136 (-0.012-0.283)	**0.259 (0.092-0.427)**	0.087 (-0.062-0.236)	0.148 (-0.024-0.320)
*(sig)*	*(0.071)*	***(0.003)***	*(0.249)*	*(0.092)*
*R*^*2*^	*0.018*		*0.007*	
**Interview score**^**#**^				
Beta (95% CIs)	**0.155 (0.012-0.297)**	**0.148 (0.006-0.291)**	0.131 (-0.013-0.274)	0.119 (-0.026-0.264)
*(sig)*	***(0.034)***	***(0.041)***	*(0.074)*	*(0.107)*
*R*^*2*^	*0.024*		*0.017*	

*All pre-admission and course performance measures standardised as z-scores.

^#^Honours & Commendation and interview scores transformed to reduce skew prior to standardisation.

^Adjusted for gender, age, ethnicity and deprivation. Note that R^2^ is not included for the adjusted models, since our focus is on the variance explained by the pre-admission measures, not the additional variance explained by gender, age, ethnicity and deprivation.

UKCAT total was strongly associated with both composite performance scores, explaining around 5% of the variance in the EPM and 6% of that in H&C. Both UKCAT verbal and quantitative reasoning were also significantly associated with the composite scores. However UKCAT decision analysis was related only to H&C and only in unadjusted analysis while UKCAT abstract reasoning was not related to either final course outcome. TSS was associated only with EPM and only after adjustment for confounders (further analyses showed that this was due to the effects of age, with those aged over 18 at entry having lower TSS, but higher EPM scores). Interview score was also associated with EPM scores, both before and after adjustment.

Given demonstrable associations between each of the pre-admissions criteria and course performance, we next aimed to investigate the *independent* associations of UKCAT, TSS and interview score with Medical School outcomes. If, in mutually adjusted analyses, each remained associated with course performance, this would indicate that all contributed independent information to the admissions process.

Table 4 therefore shows the independent associations which each of the three pre-admissions criteria had with the composite course performance indicators, both before (upper rows) and after (lower rows) adjustment for socio-demographic confounders. In general, the bivariate associations seen between each pre-admission measure and the course outcomes remained in the mutually adjusted analyses as well. Thus, UKCAT total was independently associated with both composite measures in analyses before and after adjustment for gender, age, ethnicity and deprivation. It was the only pre-admissions measure to show an independent association with H&C. TSS was significantly associated with EPM following adjustment for socio-demographic confounders. Interview score was associated with EPM in the unadjusted model, but this relationship weakened to insignificance following adjustment for confounders. Together, the three pre-admission measures explained around 8% of the variance in both composite scores, increasing, after inclusion of gender, age, ethnicity and deprivation in the models, to 16% variance in the EPM and 12% variance in H&C.

**Table 4:** Mutually adjusted associations (multiple regression analyses) between UKCAT total, total science score and interview score and composite course performance indicators* in models with and without adjustment for confounders – beta (95% confidence intervals, significance) and R^**2**^

	**Educational performance measure**			**Honours & commendation**^**#**^		
	**Beta**	***(95% CIs)***	***(sig)***	**Beta**	***(95% CIs)***	***(sig)***
**Mutually adjusted**						
UKCAT – Total score	**0.209**	***(0.065-0.352)***	***(0.005)***	**0.250**	***(0.106-0.394)***	***(0.001)***
Total Science Score	0.098	*(-0.047-0.243)*	*(0.185)*	0.049	*(-0.096-0.195)*	*(0.505)*
Interview score^#^	**0.152**	***(0.010-0.295)***	***(0.037)***	0.128	*(-0.015-0.272)*	*(0.079)*
*Model R*^*2*^	*0.081*			*0.082*		
**Mutually adjusted and also adjusted for gender, age, ethnicity & deprivation**						
UKCAT – Total score	**0.209**	***(0.065-0.353)***	***(0.005)***	**0.218**	***(0.070-0.366)***	***(0.004)***
Total science score	**0.224**	***(0.057-0.391)***	***(0.009)***	0.117	*(-0.055-0.289)*	*(0.183)*
Interview score^#^	0.125	*(-0.018-0.267)*	*(0.086)*	0.105	*(-0.042-0.252)*	*(0.159)*
*Model R*^*2*^	*0.162*			*0.117*		

*All pre-admission and course performance measures standardised as z-scores.

^#^Honours & Commendation and interview scores transformed to reduce skew prior to standardisation.

### Associations between pre-admissions measures and years 1 and 5 course outcomes

Our final secondary objective was to examine the relationships between the pre-admissions measures and the individual years 1 and 5 course outcomes. Equivalent analyses to those conducted in respect of the composite indicators showed significant associations between all pre-admissions measures (except UKCAT abstract reasoning) and the year 1 written exam, but few or none with the other year 1 performance indicators (Additional file 3: Table S2). UKCAT total generally showed the strongest associations with written exam performance. There were almost no significant associations between any pre-admissions measure and the year 5 exam results.

In mutually adjusted analysis including UKCAT total, TSS and interview score and adjusted for confounders (Additional file 4: Table S3, lower rows), all three pre-admissions measures were associated with year 1 written exam, none with year 1 Medical Independent Learning Exercise (an essay to assess independent learning and critical thinking abilities) and only UKCAT with year 1 coursework. Of the two year 5 performance indicators, only TSS was associated with the written exam and no pre-admissions measure was associated with the Objective Structured Clinical Examination (OSCE).

In identical mutually adjusted analysis (not shown) in respect of the years 2 and 3 course outcomes, the only significant associations found were between UKCAT and the year 2 written exam and OSCE, and between TSS and the written exam in both years.

## Discussion

This study reports on the predictive validity of the admissions criteria used at the University of Glasgow Medical School for performance of the first cohort of students admitted after adoption of the UKCAT in 2007. Our primary objective was to examine the associations which the UKCAT had with final (composite) course outcomes, relative to other pre-admissions criteria (TSS and pre-admissions interview scores). This is the first full cohort study to examine associations between the UKCAT and final outcome measures available at the undergraduate level.

UKCAT (total and its verbal and quantitative reasoning sub-scores) showed stronger relationships than did either TSS or interview scores with the final course outcomes. These associations between UKCAT and course performance were largely unaffected by adjustment for potentially important confounders (gender, age, ethnicity and deprivation), which is in line with the stated aims of UKCAT to remove bias from the admissions process. In mutually adjusted models, including confounders, both UKCAT total and TSS showed a significant, independent relationship with the composite EPM score that prioritises the allocation of training jobs upon graduation. However only UKCAT total score was able to significantly predict the final ranking based on overall performance in the course (H&C).

A secondary objective was to examine associations between the pre-admissions criteria and years 1 and 5 course performance indicators. Significant independent relationships between all three pre-admissions measures and the year 1 suggest that although UKCAT was most predictive of the final course outcome, each preadmissions measure might on its own right contribute to student selection.

Our findings in respect of associations between UKCAT and Glasgow Medical School performance differ from early studies from Aberdeen and Dundee [24] which found no correlation between the UKCAT scores and performance in year 1. However, they are consistent with reports from Newcastle [25], Nottingham [23] and the recent UKCAT-12 study [2] reporting a weak but significant association between UKCAT and performance in the early years. Most importantly, the association that we find between UKCAT and final ranking (H&C), further strengthens a growing body of evidence that the aptitude test adds value to the admissions process beyond the traditional interview scores and school performance indicators.

Our analysis found less socio-demographic variation in UKCAT than in TSS. Somewhat surprisingly, there was no significant socio-demographic variation in interview scores, perhaps because among this cohort, all of whom had, by definition, ‘passed’ their interview, a large proportion had similarly high scores. The analysis also found little or no relationship between UKCAT and either of the two other pre-admissions criteria, suggesting that they are measuring different dimensions.

It remains a point of discussion however, whether UKCAT is a reliable predictor of clinical aptitude in its current form. Although the Nottingham study [23] reported stronger associations with clinical course marks, both the UKCAT-12 study and the one from Newcastle Medical School [25], like the present report, found that the main associations were with knowledge based written examinations. On the other hand, the absence of significant correlations with socio-demographic and individual measures such as gender and parental higher education shows that, unlike TSS which is given high weighting in the admissions process, the aptitude test does not measurably suffer from bias favouring particular groups. This adds to the evidence base that UKCAT helps the widening participation agenda, as reported by the Durham group [20] and lends some credit to the test, which aims to make selection to medicine fairer.

It is important to acknowledge that although the variance in course performance explained by UKCAT was generally greater than that explained by either the TSS or interview scores, it is still relatively modest. It is also important to note that the purpose of the UKCAT (like TSS and interview score) is to aid in the selection of students to Medical and Dental Schools. The assumption of our study, along with all similar studies, is that the predictive power of any pre-admissions measure for student *undergraduate performance* is an indicator of its effectiveness at selecting *the best applicants*. It is possible that this assumption is incorrect. As UKCAT was originally thought to test non-cognitive skills and clinical aptitudes it is right that its success should be measured particularly against these parameters; any progress in developing the test to address these is highly anticipated. The recent addition of the Situational Judgment Test (SJT) component to the test suite, aiming to assess personality and motivation for medicine, is designed to address this need. Planned validity studies for this new component (personal communication), will further contribute to answering the question as to what is the best tool for assessing suitability for the profession.

This study, inevitably, has some inherent limitations, as we analysed only one full cohort’s results, which represents a relatively small sample size, and the particular curriculum design followed at Glasgow Medical School by the cohort admitted in 2007. In addition, only those reaching the threshold level of the UKCAT, TSS and interview scores were admitted to study at Glasgow and we do not have the means of tracking those who were not successful in entering our Medical School but may have been accepted elsewhere. We have shown a clear association of UKCAT scores with the final outcomes of one cohort, but further research is required to know whether the findings can be generalised across various Medical Schools, irrespective of teaching methods and course content. It might be beneficial to look at the combined data with other schools, however a clear advantage to a study based on only one Medical School is a lack of potential confounding factors from different curricula and teaching methods. Whether UKCAT exhibits similar predictive value irrespective of such factors will ultimately determine the utility of the test, and inform decisions in respect of its continued use alongside more traditional methods in the admissions process.

## Conclusions

The current study of a single cohort of Glasgow graduates represents the first attempt to correlate UKCAT scores with final course outcomes. It shows that the test predicts final composite scores that in turn determine the best training and career prospects upon graduation. UKCAT, school science achievements and pre-admission interview score each predicted early (year 1) written exam performance, supporting use of a combination of the various existing student selection measures. However, UKCAT was the only pre-admission measure to independently predict the final Honours and Commendation score representing students’ final course performance ranking.

### Ethical approval

Ethical approval for this study was granted by the University’s School of Medicine Research Ethics Committee.

## Abbreviations

OSCE: Objective Structured Clinical Examination; TSS: Total science score; UKCAT: United Kingdom clinical aptitude test; SJT: Situational judgment Test; EPM: Educational performance measure; H&C: Honours and Commendation.

## Competing interests

The University of Glasgow is a member of the UKCAT Consortium, but the authors in this paper have no competing interest.

## Authors’ contributions

NS had the original idea for the study, collected and collated the data and wrote the majority of the paper. JMcC, HS and NS analysed the data. HS contributed to sections of the paper. AB was responsible for acquisition of students’ demographics data. All authors read and approved the final manuscript.

## Authors’ information

All contributors are based at the University of Glasgow. NANA SARTANIA, PhD, M Ed, is a University Teacher and Deputy Head of Admissions in the Medical School; JOHN McCLURE, PhD, is a Lecturer in Statistics in the Institute of Cardiovascular and Medical Sciences, HELEN SWEETING, PhD, is a Senior Investigator Scientist in the MRC/CSO Social and Public Health Sciences Unit, and ALISON BROWITT, is a Research Associate in the Recruitment and International Office.

## Pre-publication history

The pre-publication history for this paper can be accessed here:

http://www.biomedcentral.com/1472-6920/14/116/prepub

## Supplementary Material

Additional file 1 MBChB interview scoring system. Click here for file

Additional file 2: Table S1 Significance of associations (chi-square) between socio-demographic measures. Click here for file

Additional file 3: Table S2 Bivariate associations (multiple regression analyses) between each pre-admission measure and years 1 and 5 course performance indicators* in models with and without adjustment for confounders – beta (significance) and R^2^ of unadjusted model. Click here for file

Additional file 4: Table S3 Mutually adjusted associations (multiple regression analyses) between UKCAT total, total science score and interview score and years 1 and 5 course outcomes* in models with and without adjustment for confounders – beta (significance) and model R^2^. Click here for file
