# Supplementary material for: Predictive power of UKCAT and other pre-admission measures for performance in a medical school in Glasgow: a cohort study
Source: BMC Med Educ. 2014 Jun 11;14:116. doi: 10.1186/1472-6920-14-116 (PMC4063234; doi:10.1186/1472-6920-14-116)
Supplement: Additional file 1 — MBChB interview scoring system. [file 1472-6920-14-116-S1.pdf]

27942

Copy

Interviewer 1

1

UNIVERSITY OF GLASGOW MEDICAL SCHOOL
MBChB INTERVIEW SCORING SYSTEM

Name: [redacted] [redacted] UCAS number: [redacted]

Date of interview: [redacted] Time of interview: [redacted]

1. Commitment to Medicine:

Demonstrate personal research into what a career in medicine entails

Provide evidence of attempts to experience the work of a doctor (bear in mind that not all applicants have the opportunity to do this)

Demonstrates knowledge of current issue in medical science

	1	2	3	4	5
Score (please shade ONE bubble)	○	○	○	○	○

2. Understanding of Core Qualities of a Doctor:

Demonstrate an understanding that patient care is the primary duty of a doctor

Demonstrate good communication skills and listening skills

Evidence of concern for the welfare of other (humanity)

Personal Qualities of honesty, integrity, and ability to recognise own limitation

	1	2	3	4	5
Score (please shade ONE bubble)	○	○	○	○	○

3. Team Work/Other Interests:

Understanding of how to work as part of a team

Evidence of endeavours in non academic areas

	1	2	3	4	5
Score (please shade ONE bubble)	○	○	○	○	○

4. Knowledge of Glasgow Curriculum:

Understanding of PBL

Other areas of curriculum eg clinical skills, VS, SSCs

Demonstrate more than just what is on website

	1	2	3	4	5
Score (please shade ONE bubble)	○	○	○	○	○

5. Overall:

All things considered, this candidate has the potential to cope well with a medical education and career.

	1	2	3	4	5
Score (please shade ONE bubble)	○	○	○	○	○

Interviewer's signature:

Interviewer's name: [redacted]
