# Supplementary material for: Predictive power of UKCAT and other pre-admission measures for performance in a medical school in Glasgow: a cohort study
Source: BMC Med Educ. 2014 Jun 11;14:116. doi: 10.1186/1472-6920-14-116 (PMC4063234; doi:10.1186/1472-6920-14-116)
Supplement: Additional file 2: Table S1 — Significance of associations (chi-square) between socio-demographic measures. [file 1472-6920-14-116-S2.docx]

**Additional file 2**

**Supplementary Table 1: Significance of associations (chi-square) between socio-demographic measures**

|  |  |  |  |  |  |  |
| --- | --- | --- | --- | --- | --- | --- |
|  | **Age** | **Ethnicity** | **Deprivation** | **Parental Higher Education** | **School Higher Education participation** | **Graduate** |
| **Gender** | *p = 0.382* | *p = 0.424* | *p = 0.174* | *p = 0.554* | *p = 0.423* | *p = 0.313* |
| **Age** |  | *p = 0.209* | *p = 0.278* | ***p = 0.007 ^a^*** | *p = 0.253* | ***p = 0.000 ^b^*** |
| **Ethnicity** |  |  | *p = 0.116* | *p = 0.733* | ***p = 0.000 ^c^*** | *p = 0.595* |
| **Deprivation** |  |  |  | ***p = 0.021 ^d^*** | ***p = 0.000 ^e^*** | *p = 0.155* |
| **Parental higher education** |  |  |  |  | *p = 0.396* | ***p = 0.000 ^f^*** |
| **School higher education participation** |  |  |  |  |  | *p = 0.053 ^g^* |
|  |  |  |  |  |  |  |

^a^ Those with parents with higher education more likely to be 18 or younger.

^b^ Graduates more likely to be older.

^c^ Those of non-white ethnicity more likely to be from rest of UK or overseas schools.

^d^ Those with parents with higher education more likely to be in less deprived or missing deprivation categories.

^e^ Those from Scottish schools with high levels of participation in higher education more likely to be from less deprived areas, those from Scottish schools with low levels of participation more likely to be from more deprived areas, those from schools in the rest of UK or overseas more likely to be in the missing deprivation category.

^f^ Those with parents with higher education more likely to be non-graduates at admission.

^g^ Graduates at admission more likely to come from Scottish schools with low levels of participation in higher education.
