# Supplementary material for: Predictive power of UKCAT and other pre-admission measures for performance in a medical school in Glasgow: a cohort study
Source: BMC Med Educ. 2014 Jun 11;14:116. doi: 10.1186/1472-6920-14-116 (PMC4063234; doi:10.1186/1472-6920-14-116)
Supplement: Additional file 3: Table S2 — Bivariate associations (multiple regression analyses) between each pre-admission measure and years 1 and 5 course performance indicators* in models with and without adjustment for confounders – beta (significance) and R2 of unadjusted model. [file 1472-6920-14-116-S3.docx]

**Additional file 3**

**Supplementary Table 2: Bivariate associations (multiple regression analyses) between each pre-admission measure and years 1 and 5 course performance indicators* in models with and without adjustment for confounders – beta (significance) and R^2^ of unadjusted model**

|  |  | | | | | |  | | | |
| --- | --- | --- | --- | --- | --- | --- | --- | --- | --- | --- |
|  | **Year 1** | | | | | | **Year 5** | | | |
|  | **Written Exam** | | **MILE** | | **Coursework** | | **Written Exam^#^** | | **OSCE** | |
|  | **Unadj** | **Adj^** | **Unadj** | **Adj^** | **Unadj** | **Adj^** | **Unadj** | **Adj^** | **Unadj** | **Adj^** |
|  |  |  |  |  |  |  |  |  |  |  |
| **UKCAT – Total score** |  |  |  |  |  |  |  |  |  |  |
| Beta | **0.252** | **0.257** | 0.059 | 0.110 | **0.149** | **0.182** | **0.187** | 0.136 | -0.008 | 0.035 |
| *(sig)* | ***(0.000)*** | ***(0.001)*** | *(0.419)* | *(0.150)* | ***(0.041)*** | ***(0.016)*** | ***(0.011)*** | *(0.072)* | *(0.919)* | *(0.642)* |
| *R^2^* | *0.064* |  | *0.004* |  | *0.022* |  | *0.035* |  | *0.000* |  |
| **UKCAT – Verbal reasoning** |  |  |  |  |  |  |  |  |  |  |
| Beta | **0.174** | **0.193** | 0.021 | 0.049 | 0.066 | 0.084 | **0.145** | 0.100 | 0.053 | 0.060 |
| *(sig)* | ***(0.017)*** | ***(0.009)*** | *(0.775)* | *(0.513)* | *(0.367)* | *(0.261)* | ***(0.048)*** | *(0.175)* | *(0.475)* | *(0.411)* |
| *R^2^* | *0.031* |  | *0.000* |  | *0.004* |  | *0.021* |  | *0.003* |  |
| **UKCAT – Quantitative reasoning** |  |  |  |  |  |  |  |  |  |  |
| Beta | **0.197** | **0.201** | -0.067 | 0.001 | 0.109 | **0.182** | **0.155** | 0.126 | 0.043 | 0.123 |
| *(sig)* | ***(0.007)*** | ***(0.009)*** | *(0.363)* | *(0.985)* | *(0.136)* | ***(0.019)*** | ***(0.034)*** | *(0.102)* | *(0.562)* | *(0.105)* |
| *R^2^* | *0.039* |  | *0.004* |  | *0.012* |  | *0.025* |  | *0.002* |  |
| **UKCAT – Decision analysis** |  |  |  |  |  |  |  |  |  |  |
| Beta | **0.172** | **0.154** | 0.097 | 0.113 | 0.112 | 0.103 | 0.093 | 0.055 | -0.067 | -0.058 |
| *(sig)* | ***(0.018)*** | ***(0.039)*** | *(0.188)* | *(0.130)* | *(0.126)* | *(0.166)* | *(0.212)* | *(0.460)* | *(0.369)* | *(0.428)* |
| *R^2^* | *0.030* |  | *0.009* |  | *0.013* |  | *0.009* |  | *0.004* |  |
| **UKCAT – Abstract reasoning** |  |  |  |  |  |  |  |  |  |  |
| Beta | 0.130 | 0.130 | 0.100 | 0.110 | 0.112 | 0.119 | 0.112 | 0.083 | -0.042 | -0.013 |
| *(sig)* | *(0.074)* | *(0.077)* | *(0.174)* | *(0.135)* | *(0.125)* | *(0.105)* | *(0.127)* | *(0.260)* | *(0.572)* | *(0.859)* |
| *R^2^* | *0.017* |  | *0.010* |  | *0.013* |  | *0.013* |  | *0.002* |  |
| **Total Science Score** |  |  |  |  |  |  |  |  |  |  |
| Beta | **0.219** | **0.289** | -0.106 | -0.059 | -0.056 | 0.035 | 0.131 | **0.198** | **-0.236** | -0.112 |
| *(sig)* | ***(0.003)*** | ***(0.001)*** | *(0.152)* | *(0.491)* | *(0.450)* | *(0.680)* | *(0.082)* | ***(0.022)*** | ***(0.002)*** | *(0.190)* |
| *R^2^* | *0.047* |  | *0.011* |  | *0.003* |  | *0.017* |  | *0.054* |  |
| **Interview score^#^** |  |  |  |  |  |  |  |  |  |  |
| Beta | **0.165** | **0.181** | -0.024 | -0.008 | -0.018 | -0.004 | 0.042 | 0.032 | 0.114 | 0.097 |
| *(sig)* | ***(0.023)*** | ***(0.014)*** | *(0.740)* | *(0.918)* | *(0.809)* | *(0.959)* | *(0.565)* | *(0.666)* | *(0.118)* | *(0.177)* |
| *R^2^* | *0.028* |  | *0.001* |  | *0.000* |  | *0.002* |  | *0.013* |  |
|  |  |  |  |  |  |  |  |  |  |  |

* All pre-admission and course performance measures standardised as z-scores.

^#^ Year 5 written exam and interview scores transformed to reduce skew prior to standardisation.

^ Adjusted for gender, age, ethnicity and deprivation. Note that R^2^ is not included for the adjusted models, since our focus is on the variance explained by the pre-admission measures, not the additional variance explained by gender, age, ethnicity and deprivation.

MILE = Medical Independent Learning Exercise; OSCE = Objective Structured Clinical Examination.
