# Supplementary material for: Predictive power of UKCAT and other pre-admission measures for performance in a medical school in Glasgow: a cohort study
Source: BMC Med Educ. 2014 Jun 11;14:116. doi: 10.1186/1472-6920-14-116 (PMC4063234; doi:10.1186/1472-6920-14-116)
Supplement: Additional file 4: Table S3 — Mutually adjusted associations (multiple regression analyses) between UKCAT total, total science score and interview score and years 1 and 5 course outcomes* in models with and without adjustment for confounders – beta (significance) and model R2. [file 1472-6920-14-116-S4.doc]

**Additional file 4**

**Supplementary Table 3: Mutually adjusted associations (multiple regression analyses) between UKCAT total, total science score and interview score and years 1 and 5 course outcomes* in models with and without adjustment for confounders – beta (significance) and model R^2^**

|  |  | | | | | |  | | | |
| --- | --- | --- | --- | --- | --- | --- | --- | --- | --- | --- |
|  | **Year 1** | | | | | | **Year 5** | | | |
|  | **Written exam** | | **MILE** | | **Coursework** | | **Written exam^#^** | | **OSCE** | |
|  | **Beta** | ***(sig)*** | **Beta** | ***(sig)*** | **Beta** | ***(sig)*** | **Beta** | ***(sig)*** | **Beta** | ***(sig)*** |
| **Mutually adjusted** |  |  |  |  |  |  |  |  |  |  |
| UKCAT –  Total score | **0.241** | ***(0.001)*** | 0.061 | *(0.414)* | 0.147 | *(0.050)* | **0.161** | ***(0.031)*** | 0.023 | *(0.757)* |
| Total Science Score | **0.174** | ***(0.015)*** | -0.113 | *(0.132)* | -0.071 | *(0.344)* | 0.112 | *(0.136)* | **-0.258** | ***(0.001)*** |
| Interview score^#^ | **0.158** | ***(0.027)*** | 0.013 | *(0.866)* | 0.007 | *(0.921)* | 0.045 | *(0.542)* | 0.154 | *(0.035)* |
| *Model R^2^* | *0.126* | | *0.015* | | *0.024* | | *0.044* | | *0.078* | |
| **Mutually adjusted and also adjusted for gender, age, ethnicity &deprivation** |  |  |  |  |  |  |  |  |  |  |
| UKCAT –  Total score | **0.252** | ***(0.001)*** | 0.109 | *(0.153)* | **0.169** | ***(0.027)*** | 0.119 | *(0.115)* | 0.040 | *(0.597)* |
| Total Science Score | **0.239** | ***(0.004)*** | -0.073 | *(0.402)* | 0.022 | *(0.799)* | **0.189** | ***(0.031)*** | -0.143 | *(0.100)* |
| Interview score^#^ | **0.160** | ***(0.027)*** | 0.035 | *(0.643)* | 0.015 | *(0.843)* | 0.018 | *(0.809)* | 0.131 | *(0.078)* |
| *Model R^2^* | *0.176* | | *0.065* | | *0.078* | | *0.104* | | *0.126* | |
|  |  |  |  |  |  |  |  |  |  |  |

* All pre-admission and course performance measures standardised as z-scores.

^#^ Year 5 written exam and interview scores transformed to reduce skew prior to standardisation.

MILE = Medical Independent Learning Exercise; OSCE = Objective Structured Clinical Examination.
